# The Treatment of Burns

**Published:** 1899-01

**Authors:** M. M. Pool

**Affiliations:** El Campo, Texas


### The Treatment of Burns.

BY M. M. POOL, M. D., EL CAMPO, TEXAS.

Read at Austin District Medical Society, September 29, 1898.

In presenting a paper under this title, I do not pretend to offer any new treatment, but simply wish to summarize.

Of the surgical cases to which the general practitioner is called, burns and scalds are undoubtedly the most common, and the standard text-books on surgery say very little about the treatment of minor lesions, giving most of their space to those requiring surgical interference, notwithstanding, when called to a case of burn, we are more than apt to find lesions of every degree, hence, the treatment of all will be considered at the same time.

The first indication is to relieve pain, and prevent collapse or modify the intensity of shock. For the relief of pain, exclude air by oils, very fine powders, immersion in water, or by the use of morphine or its congeners.

Shock should be treated by judicious stimulation with whiskey or brandy by enema, also from one-fourth to one-half pint of warm water, to which has been added one egg and from one-half to one ounce of turpentine. One pint of strong black coffee, with one-twentieth grain strichnia by enema, is also fine. This should be repeated after a while, and from twenty to thirty minims of digitalis with 1-100 grain of atropine as indicated.

The second indication is to prevent infection, and for this antisepsis should be maintained, but great care should be used in the employment of such antiseptics as carbolic acid and bichloride of mercury, as absorption occurs very readily from burned surfaces.

The third indication being to hasten repair, may be facilitated by transplantation of skin by grafting, sliding, or transplantation in mass.

In giving the treatment of burns, I shall not attempt to enumerate the long list of remedies that have been recommended from time to time, but those I deem most efficacious.

The patient should be handled with the utmost care, all blebs opened as they form, and dressings changed every two or three days.

Of the medicinal agents, carron oil is perhaps the most popular dressing we have for burns, but it is a nasty, greasy mess. Salicylic acid, dr. i, to olive oil, oz. viii, is also a good application, but likewise disagreeable. A one per cent. solution of carbolic acid will relieve pain and prevent suppuration, but danger of absorption should not be forgotten. Campho-phenique is another good preparation. All of them should be applied with absorbent cotton and bandaged.

Ichthyol is excellent, and far more preferable than any of the above; it alleviates pain, reduces œdema, and promotes healing. It is used in ten per cent. aqueous solution, or ointment, and also diluted with oxide of zinc or bismuth, and used dry. Dr. Alger, Professor of Dermatology in the New York Polyclinic, says the ten per cent. solution is made much better by adding a little starch and egg albumin.

Dr. M. Paggi recommends nitrate of potash in all kinds of burns of whatever degree; he says it acts as a refrigerant (any solution up to saturation); as it becomes dissolved in the water it produces a notable lowering of the temperature of the liquid of from five to nine degrees F. If a burned hand or foot be plunged into a basin of water to which a few spoonfuls of the nitrate has been added, the pain ceases rapidly; if the water becomes slightly heated, the pain returns, but it is allayed as soon as a fresh quantity of the salt is added. This bath, which is prolonged from two to three hours, may bring about the definite disappearance of the pain, and even prevent the production of blisters. The application of compresses also exercises the same influence. By this means the pain is allayed and cicatrization takes place without delay.

Quoting Dr. McInnis, of Canada, "Spirits of turpentine applied to burns of the first, second or third degree will almost at once relieve pain. The burn will heal more rapidly than by any other treatment." He uses it as follows: Wrap a thin layer of absorbent cotton over the burn, saturate it with turpentine and bandage, and as it evaporates apply more to the dressing. Open blebs on the second or third day. Protect the unburned skin from the turpentine.

Dr. Thiery, of Paris, recommends picric acid, as follows: Immerse the part in a solution of one part acid to sixty parts water five minutes, then wrap the part with wadding, protecting it, if excoriated, by iodoform gauze.

Dr. Alger, of New York, says: "I have gradually come to consider picric acid the best all-around dressing for burns. It is a fair antiseptic, a good oxidizing agent, and has to a very high degree the power of coagulating albumin. I have used it in a good many forms, but have found the combination with citric acid by far the most satisfactory. R. Picric acid, 10 parts; citric acid, 20 parts; aquæ, *ad.* 100 parts. The fluid should be mopped on freely, care being taken that it reaches the interior of every vesicle. Picric acid alone is a rather weak acid, and coagulates albumin but poorly in an alkaline medium. The citric acid acidulates the alkaline exudate, which the picric acid promptly converts into an antiseptic coagulum, capable of excluding the air and resisting infection. This combination, after a momentary smarting, relieves the pain more quickly and completely than anything I have ever tried. After the excess of fluid has drained off, the part may be covered with soft gauze and not disturbed for several days.

After the first dressing, it should be reapplied every two or three days only to those areas where exudative fluid has formed. It makes a clean, comfortable dressing, and I have never observed any toxic effect.

Dr. Walther, of Paris, reports two patients treated by Dr. Latouche with saturated solution of picric acid and an ointment one part acid to ten parts vaseline (each patient getting 20 grams of the acid) caused deplorable results, each had violent pain, incessant vomiting, active colics, yellowish diarrhœic motions, tendency to coma, general icterus, and scanty, black non-albuminous urine, heavily charged with picric acid, which lasted two to eight days respectively, but the wounds healed in about twenty-five days. They were of the first and second degrees.

The injection of artificial serum by Tomasoli, as a method of preventing death from extensive burns, has been verified by experiment upon animals. He says, if serum be taken from one scalded animal (not treated by saline injection), of if from the flesh of one of them an extract be made, and a definite quantity of this serum or extract, proportionate to the weight of the animal experimented upon, be injected into a healthy one, it will die. On the other hand, if an animal in practically the same condition as the other one, be injected with artificial serum immediately after receiving a lethel dose of the extract, this one will not die.

The serum employed is a solution of sodium chloride and sodium bicarbonate. From 250 to 500 grams daily have been used for a period of three weeks, with happy results.

As no deaths have been caused by picric acid, I much prefer it and ichthyol in burns of every degree.

Complications are to be treated on general principles. For the treatment of cicatrices resulting from extensive burns, I refer you to the able authorities on surgery.

